# CDKB2 is involved in mitosis and DNA damage response in rice

**DOI:** 10.1111/j.1365-313X.2011.04847.x

**Published:** 2011-12-15

**Authors:** Masaki Endo, Shigeki Nakayama, Chikage Umeda-Hara, Namie Ohtsuki, Hiroaki Saika, Masaaki Umeda, Seiichi Toki

**Affiliations:** 1 Plant Genome Engineering Research Unit, Agrogenomics Research Center, National Institute of Agrobiological Sciences2-1-2 Kannondai, Tsukuba, Ibaraki 305-8602, Japan; 2 Graduate School of Biological Sciences, Nara Institute of Science and TechnologyTakayama 8916-5, Ikoma, Nara 630-0101, Japan

**Keywords:** CDKB2, DNA damage, rice, endomitosis, ploidy, cell cycle

## Abstract

DNA damage checkpoints delay mitotic cell-cycle progression in response to DNA stress, stalling the cell cycle to allow time for repair. CDKB is a plant-specific cyclin-dependent kinase (CDK) that is required for the G_2_/M transition of the cell cycle. In Arabidopsis, DNA damage leads the degradation of CDKB2, and the subsequent G_2_ arrest gives cells time to repair damaged DNA. G_2_ arrest also triggers transition from the mitotic cycle to endoreduplication, leading to the presence of polyploid cells in many tissues. In contrast, in rice (*Oryza sativa*), polyploid cells are found only in the endosperm. It was unclear whether endoreduplication contributes to alleviating DNA damage in rice *(Oryza sativa)*. Here, we show that DNA damage neither down-regulates *Orysa*;CDKB2;1 nor induces endoreduplication in rice. Furthermore, we found increased levels of *Orysa*;CDKB2;1 protein upon DNA damage. These results suggest that CDKB2 functions differently in Arabidopsis and rice in response to DNA damage. Arabidopsis may adopt endoreduplication as a survival strategy under genotoxic stress conditions, but rice may enhance DNA repair capacity upon genotoxic stress. In addition, polyploid cells due to endomitosis were present in *CDKB2;1* knockdown rice, suggesting an important role for *Orysa;*CDKB2;1 during mitosis.

## Introduction

Repairing DNA damage in a timely fashion is essential to maintaining genome integrity. DNA damage triggers activation of the DNA repair machinery and delay or arrest of cell-cycle progression to allow sufficient time for damaged DNA to be repaired before proceeding to mitosis. DNA damage signals are transmitted via several proteins, suppressing the activity of cyclin-dependent kinase (CDK) to arrest the cell cycle (Sancar *et al.*, 2004). Like animals, plants have multiple CDK-related protein kinases, which are classified into six types: CDKA–F (Joubès *et al.*, 2000). A-type CDK (CDKA) is closely related to yeast Cdc2/Cdc28, and is expressed throughout the cell cycle (Colasanti *et al.*, 1991; Ferreira *et al.*, 1991; Hirt *et al.*, 1991, 1993; Fobert *et al.*, 1996). However, B-type CDKs (CDKBs) are plant-specific, and are further classified into two sub-types: CDKB1 and CDKB2. Expression of CDKBs is under strict cell-cycle control; CDKB1 is expressed from late S to M phase, while CDKB2 is expressed from G_2_ to M phase, as confirmed in experiments in alfalfa (*Medicago sativa*) (Magyar *et al.*, 1997), rice (*Oryza sativa*) (Umeda *et al.*, 1999b), tobacco (*Nicotiana tabacum*) (Porceddu *et al.*, 2001) and Arabidopsis (Menges *et al.*, 2005).

In Arabidopsis, DNA double-strand breaks (DSBs) have been reported to enhance endoreduplication (Ramirez-Parra and Gutierrez, 2007; Adachi *et al.*, 2011). Cells undergoing endoreduplication replicate chromosomal DNA without intervening mitoses, and the resulting larger, higher-ploidy nucleus is often associated with an increase in cell size. A major component of the switch to endoreduplication is prevention of mitosis by reduction of mitotic CDK activity to a level that does not initiate mitosis but is able to drive replication of DNA. Consistent with this, reduced expression of CDK and cyclin B was seen during endoreduplication in trichome, epidermal and mesophyll cells of Arabidopsis leaves (Schnittger *et al.*, 2003; Verkest *et al.*, 2005). Endoreduplication often occurs in cell types that undergo specialized differentiation, such as hair cells and xylem cells, or in plant cell types that have high metabolic activity, such as endosperm and embryo suspensor cells. Plants use endoreduplication as a bypass pathway to avoid cell-cycle arrest or cell death by DNA damage, because endoreduplicated cells remain where they were generated, and thus contribute to organogenesis as a constituent of tissues.

In contrast to Arabidopsis, and other plants in which polyploid cells are found in many tissues, polyploid cells are found only in the endosperm in rice. Furthermore, the effect of DNA damage on cell cycle and ploidy in rice has not yet been explored. Thus, it is of great interest to determine how rice responds to damaged DNA in terms of the cell cycle. We are currently analyzing the effect of DNA damage on ploidy and CDKB2 expression in rice. Arabidopsis has two B2-type CDKs: CDKB2;1 and CDKB2;2. Both genes show a peak in expression during the G_2_/M phase transition of the cell cycle (Menges *et al.*, 2003). DNA damage degrades *Arath;*CDKB2;1 (Adachi *et al.*, 2011), and *Arath;*CDKB2;1 down-regulation is associated with endoreduplication (Andersen *et al.*, 2008). In contrast, rice has a single gene (*CDKB2;1*) encoding CDKB2 (Umeda *et al.*, 1999b). Transcription of *Orysa;CDKB2;1* is abundant during progress from G_2_ to M phase (Umeda *et al.*, 1999b).

Here, we reveal that DNA damage neither induces endoreduplication nor down-regulates *Orysa*;CDKB2;1 expression. Instead, *Orysa*;CDKB2;1 accumulates in response to DNA damage. As in Arabidopsis, an *Orysa;CDKB2;1* knockdown mutant showed polyploidy in rice calli, but this was shown to be due to endomitosis rather than endoreduplication.

## Results and Discussion

### DSBs do not induce polyploidy in rice

In rice plants, mitotic cells are limited exclusively to the root, shoot and intercalary meristems, thus we used callus cells cultured on solid medium to investigate the effects of DNA damage on rice cell-cycle progression. We first monitored the extent of DNA DSBs in the genomic DNA of rice calli irradiated with various doses of X-rays using the comet assay (Menke *et al.*, 2001). In this assay, the ‘tail moment’ represents the level of DNA damage.

**Figure 1:**
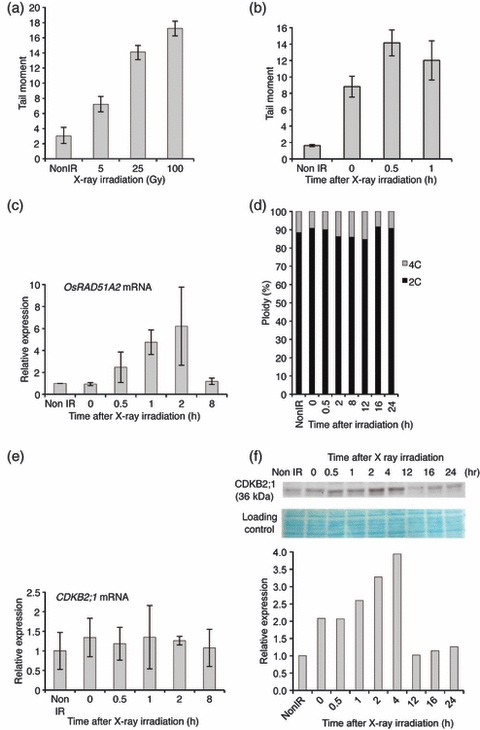
DNA damage response in rice calli following X-ray irradiation. (a) Quantitative analysis of DSBs by the comet assay. Wild-type rice calli were irradiated with 5, 25 and 100 Gy doses of X-rays at 133 Gy h^−1^, and tail moment values were measured just after irradiation. (b) Quantification of DSBs by the comet assay at various time points after irradiation of calli with a 5 Gy dose of X-rays. (c) Transcription level of *OsRAD51A2* determined by real-time quantitative PCR. (d) Ploidy of X-ray-irradiated rice calli. The DNA content of nuclei prepared from calli was analyzed by flow cytometry at various times following irradiation. (e) Transcription levels of *CDKB2;1* determined by real-time quantitative PCR. (f) Immunological detection of CDKB2;1. Upper panel: Western blot analysis of protein extracts from non-irradiated (Non-IR) or X-ray-irradiated rice calli using antibodies against *Orysa*;CDKB2;1. Middle panel: stained membrane showing equal loading of protein samples. Lower panel: quantification of CDKB2;1 protein.

Upon Xray exposure, DNA damage increased in a dose-dependent manner (Figure 1a). Beyond a certain threshold, when DNA damage is too severe, cell viability itself is also decreased, and it becomes difficult to evaluate the biological effects precisely. Thus, we used 5 Gy as the X-ray irradiation dose. In calli irradiated with 5 Gy, DNA damage was maximal at 30 min, decreasing again at 1 h after irradiation (Figure 1b). This result shows that rice callus does not lose its DNA repair machinery following a 5 Gy dose of irradiation. To confirm that 5 Gy irradiation is sufficient to induce a DNA damage response, we analyzed the transcription level of the rice *RAD51* gene. The Rad51 protein is crucial to homologous recombination repair, and transcription of *RAD51* is drastically induced by DNA damage in Arabidopsis (Culligan *et al.*, 2006). Two Rad51 orthologs, Rad51A1 (Os11g0615800) and Rad51A2 (Os12g0497300) exist in rice. Transcription of *RAD51A2* (but not *RAD51A1*) is enhanced by DNA damage (M.E., unpublished data). *OsRAD51A2* transcription was increased by 5 Gy X-ray irradiation (Figure 1c).

Next, we analyzed ploidy levels in X-ray-irradiated rice calli to investigate the effect of DNA damage on cell-cycle progression. 2C represents G_1_ cells and 4C represents G_2_ cells. We detected no significant change in DNA ploidy distribution, and observed no 8C or 16C cells within 24 h of X-ray irradiation (Figure 1d) or later (data not shown). We also analyzed the effect of 25 and 100 Gy irradiation on ploidy but detected no polyploid cells (Figure S1a,b). From these data, we concluded that endoreduplication is not a major DNA damage response in rice. This conclusion was also supported by data obtained from bleomycin-treated calli (discussed below).

### *Orysa;CDKB2;1* is not down-regulated by DNA damage

A major component of the switch to endoreduplication is prevention of mitosis by reducing CDK activity to a level that does not initiate mitosis but is able to drive replication of DNA (for review, see John and Qi, 2008). Indeed, in Arabidopsis, knockdown of *CDKB2* led to higher nuclear DNA content than in wild-type (Andersen *et al.*, 2008), suggesting that DNA damage signaling could reduce the amount of CDKB2, resulting in enhanced endoreduplication in Arabidopsis. Furthermore, a recent study by Adachi *et al.* (2011) demonstrated this connection directly.

To analyze the relationship between DNA damage signaling and the CDKB2 expression level in rice, we analyzed transcription and protein levels of *Orysa*;CDKB2;1 after X-ray irradiation. Transcript levels of *Orysa*;*CDKB2;1* were not affected significantly by X-ray irradiation (Figure 1e), but we found increased levels of CDKB2;1 protein immediately after X-ray irradiation, and levels continued to increase for at least 4 h (Figure 1f).

**Figure 2:**
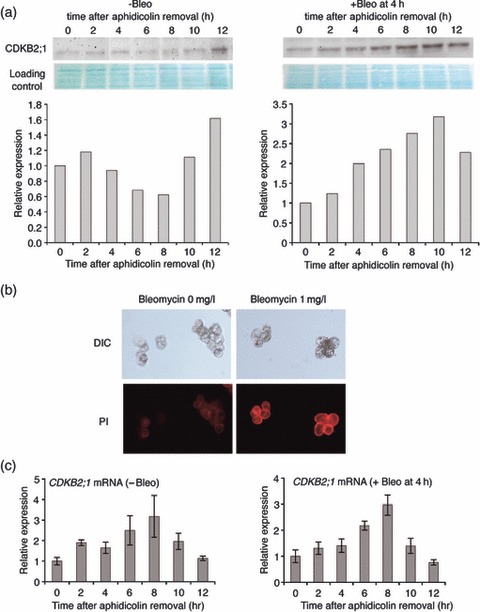
DNA damage response in rice suspension-cultured cells after bleomycin treatment. (a) Immunoblot analysis of CDKB2;1 using rice suspension-cultured cells. Suspension-cultured cells were blocked in S phase for 24 h using aphidicolin (left panel). The DNA-damaging agent bleomycin (1 mg L^−1^) was added to the culture medium 4 h after aphidicolin block removal (right panel). Crude proteins were prepared at 2 h intervals after release from the aphidicolin block. (b) PI staining of suspension-cultured cells with or without 2 h bleomycin treatment. (c) Transcription level of *CDKB2;1* determined by real-time quantitative PCR.

The expression profile of CDKB2;1 upon cell-cycle progression and DNA damage was confirmed in the rice suspension-cultured cell line OC (Baba *et al.*, 1986) using the DNA polymerase inhibitor aphidicolin (Spadari *et al.*, 1984) and the radiomimetic reagent bleomycin. Aphidicolin arrests cells at S phase; release from this block enables cells to progress synchronously through S, G_2_ and M phase. Thus, by using synchronized OC cells, we expected to observe cell cycle-dependent oscillation of CDKB2;1 expression, in addition to CDKB2;1 expression in response to DNA damage. Without bleomycin treatment, protein levels of CDKB2;1 first peaked at 2 h, decreased from 2–8 h, but started to accumulate again from 8 h after aphidicolin removal (Figure 2a, −Bleo). However, when bleomycin was added to suspension cultured cells 4 h after aphidicolin removal, protein levels of CDKB2;1 continued to increase (Figure 2a, +Bleo). We confirmed that this treatment was sufficient to induce DNA damage, which led to cell death as shown by propidium iodide (PI) staining of cells. PI is used as a marker for loss of membrane integrity and cell death, as dead cells take up PI readily, in contrast to live cells, which actively exclude it (Truernit and Haseloff, 2008). PI staining was only detected in bleomycin-treated OC cells (Figure 2b). Transcription of *CDKB2;1* did not differ much between bleomycin-treated and untreated cells (Figure 2c). Taken together, these results indicate that DNA damage does not affect transcription but instead increases the amount of CDKB2;1 at the protein level.

### Knockdown of *Orysa;CDKB2;1* induces polyploidy

**Figure 3:**
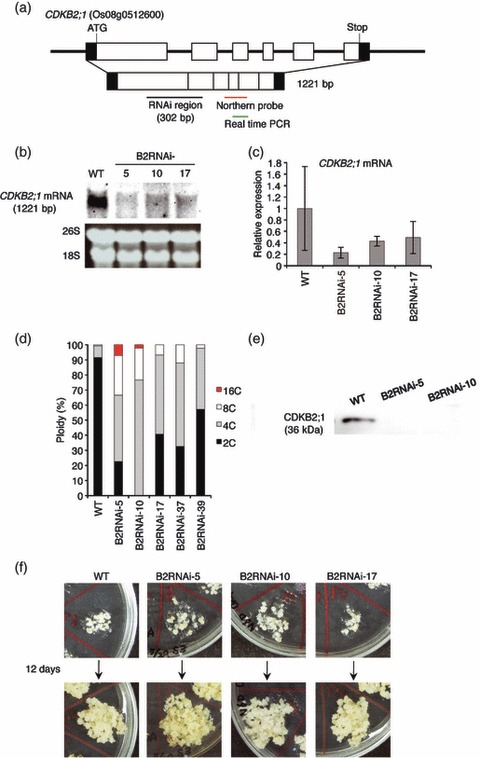
Characterization of *Orysa;CDKB2;1* knockdown calli. (a) Schematic representation of *CDKB2;1* structure and RNAi silencing target region. (b) Northern blot analysis of *CDKB2;1*. Upper panel: *OsCDKB2;1* mRNA. Lower panel: RNAs transferred to nylon membrane to visualize equal loading of total RNA in each lane. (c) Transcription level of *CDKB2;1* determined by real-time quantitative PCR. The region amplified is indicated in (a). (d) Ploidy of *CDKB2;1* knockdown rice calli (B2RNAi) grown on selection medium for 36 days. (e) Immunological detection of CDKB2;1. (f) Growth of wild-type (WT) and B2RNAi calli on N6D solid medium at 0 days (upper panel) and 12 days (lower panel) after transfer.

We next speculated that the lack of *CDKB2;1* down-regulation in response to DNA damage could explain why rice does not enter the endocycle. To test this hypothesis, we generated *CDKB2;1* constitutive knockdown mutants (B2RNAi). We cloned an RNAi construct towards an internal 302 bp region of *CDKB2;1* (Figures 3a and S2) into the pANDA vector (Miki and Shimamoto, 2004), which incorporates a maize ubiquitin promoter and intron (Christensen *et al.*, 1992), and produces high levels of trigger dsRNA in transgenic rice (Miki and Shimamoto, 2004). We confirmed decreased expression of *CDKB2;1* in B2RNAi T_0_ calli (Figure 3b,c). Despite a low level of sequence homology of this RNAi region with rice *CDKA;1*, *CDKA;2* or *CDKB1;1* (Figure S2), no statistically significant difference in transcription of *CDKA;1*, *CDKA;2* and *CDKB1;1* between wild-type and B2RNAi lines was seen (Figure S3). To show any effect of *CDKB2;1* knockdown on DNA ploidy distribution, we analyzed ploidy in B2RNAi calli by flow cytometric analysis. In B2RNAi calli, the 4C nuclei population increased drastically at the expense of the proportion of nuclei with 2C (Figure 3d). Surprisingly, 8C and 16C fractions also appeared in B2RNAi calli. We confirmed that the CDKB2;1 protein level was severely reduced in B2RNAi calli showing polyploidy (Figure 3e). Initially, we assumed that the polyploidy detected in B2RNAi calli was due to endoreduplication, because polyploid cells are known to occur in rice endosperm (Larkins *et al.*, 2001). Cells that enter endoreduplication cannot proliferate because the endocycle repeats DNA synthesis without mitosis. However, the cell proliferation of B2RNAi calli was comparable to that of wild-type calli, despite the high population of polyploid cells (Figure 3f). Given these results, we hypothesized that endomitosis rather than endoreduplication occurs in B2RNAi calli.

### The polyploidy in the *Orysa;CDKB2;1* knockdown line is due to endomitosis

**Figure 4:**
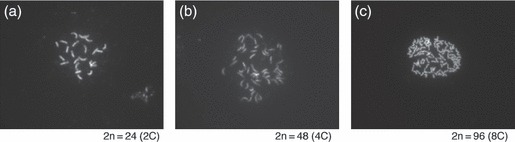
Chromosome observation of *Orysa;CDKB2;1* knockdown calli. Chromosome spreads of nuclei from *Orysa;CDKB2;1* knockdown line B2RNAi-5. Nuclei containing 24 (a), 48 (b) or 96 (c) chromosomes were found.

During endomitosis, chromosomes double and condense, and sister chromatids separate and return to the interphase state as in the mitotic cycle. As a result, chromosome numbers double in each cycle. On the other hand, endoreduplication involves an endonuclear chromosome duplication that occurs in the absence of any obvious condensation or decondensation steps (Lorz, 1947; Levan and Hauschka, 1953). Therefore, although DNA content doubles in each nucleus, chromosome number does not change in cells undergoing endoreduplication. To distinguish between endoreduplication and endomitosis, we analyzed the chromosome number of polyploid cells. We found nuclei with 24, 48 and 96 chromosomes in line B2RNAi-5 (Figure 4a–c), which harbors 2C, 4C, 8C and 16C nuclei (Figure 3d). In line B2RNAi-10, which did not contain 2C nuclei according to flow cytometric analysis (Figure 3d), we detected nuclei with 48 and 96 chromosomes but no nuclei with 24 chromosomes within eight analyzed nuclei (Figure S4). From these data, we conclude that the polyploid cells found in B2RNAi calli result from endomitosis. Transcription of *CDKB2;1* has been reported to be limited to the dividing region of rice roots (Umeda *et al.*, 1999a), and *CDKB2;1* transcripts were abundant from G_2_ to M phase but disappear when cells complete mitosis at telophase (Umeda *et al.*, 1999b). Furthermore, a CDKB2;1–GFP fusion protein was tightly associated with chromosome alignment as well as with the spindle structure during metaphase (Lee *et al.*, 2003). During telophase, this GFP signal was localized to the spindle mid-zone and the separating sister chromosomes, and then to the phragmoplast (Lee *et al.*, 2003). These observations strongly support the idea that CDKB2;1 plays a functional role in the separation of sister chromatids.

### Decreased expression of *Orysa;CDKB2;1* disturbs morphogenesis

Increased nuclear DNA content often occurs concomitantly with the onset of cell expansion (Sugimoto-Shirasu and Roberts, 2003). In order to investigate the effect of endomitosis on rice morphogenesis, we attempted to regenerate tetraploid plants from B2RNAi calli. No tetraploid plants were detected in 34 plants regenerated from 11 independent B2RNAi calli, despite the fact that these calli showed a high population of polyploid cells and proliferated well. Furthermore, root growth of some diploid regenerated plants ceased soon after regeneration (Figure S5a). We changed the medium from MS hormone-free solid medium to MS liquid medium because liquid medium is more easily absorbed and allows roots to grow without physical impedance. Despite this, these plants stopped growing and died (Figure S5b). Based on these results, we conclude that constitutive knockdown of *CDKB2;1* induces endomitosis in calli, and that it also disturbs regeneration and growth of regenerated plants.

**Figure 5:**
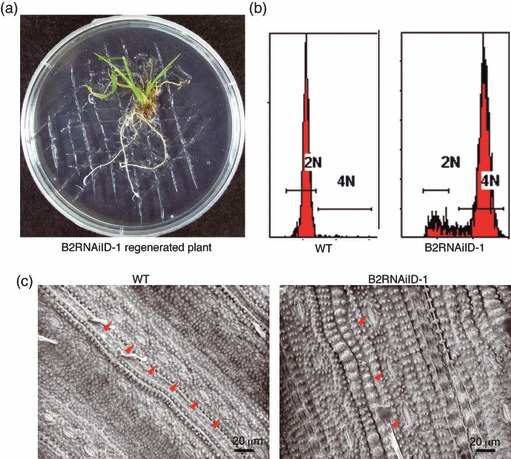
Phenotypic analysis of a regenerated plant from the *Orysa;CDKB2;1* inducible knockdown line. (a)Tetraploid rice plant regenerated from *CDKB2;1* inducible RNAi transformed callus (B2RNAiID-1). (b) Flow cytometry measurements of DNA content from leaf cells of wild-type and B2RNAiID-1. (c) Scanning electron micrographs of the epidermal leaf surface of wild-type (diploid) and B2RNAiID-1 (tetraploid) plants. Arrowheads indicate stomata.

Although we could not regenerate tetraploid rice plants from *CDKB2;1* constitutive knockdown calli (B2RNAi), we expected that tetraploid rice plants could be regenerated from calli in which endomitosis was induced temporarily. To this end, we used the XVE induction system (Zuo *et al.*, 2000) to create *CDKB2;1* knockdown plants (see Appendix S1). We observed that β-estradiol treatment increased the 4C population, and removal of β-estradiol reversed this increase in induced *CDKB2;1* RNAi-transformed calli (B2RNAiID) (Figure S6). After induction of endomitosis in B2RNAiID calli by β-estradiol treatment and transfer of these calli to regeneration medium without β-estradiol, we succeeded in obtaining a tetraploid plantlet, B2RNAiID-1 (Figure 5a,b). The files of epidermal cells in B2RNAiID-1 leaves were wider than those of wild-type, and the guard cells were significantly larger (Figure 5c). Correlation between ploidy, nuclear DNA content and cell size has been reported in Arabidopsis and many other plant species (Nagl, 1978; Galbraith *et al.*, 1991; Melaragno *et al.*, 1993). This correlation was also seen in the tetraploid rice plant generated by *CDKB2;1* inducible knockdown. Normally, epidermal cells of rice blades are arranged regularly in rows parallel to the midrib, but the files of the B2RNAiID-1 leaf epidermis were disordered. Well-organized cell division at the shoot apical meristem is necessary for orderly phyllotaxis. Rice *flattened shoot meristem* (*fsm*) mutants lacking the p150 subunit of CAF-1, which is tightly associated with cell-cycle progression, showed a defect in maintenance of the shoot apical meristem as well as irregular cell files (Abe *et al.*, 2008). Therefore, we speculated that the morphological aberration detected in B2RNAiID plants was caused by non-cooperative cell division due to leaky expression of *CDKB2;1* RNAi during regeneration. All of these results suggest that expression of CDKB2;1 must be under strict control in order for normal development of rice to occur.

### Decreased *Orysa;CDKB2;1* expression induces DNA damage hypersensitivity

**Figure 6:**
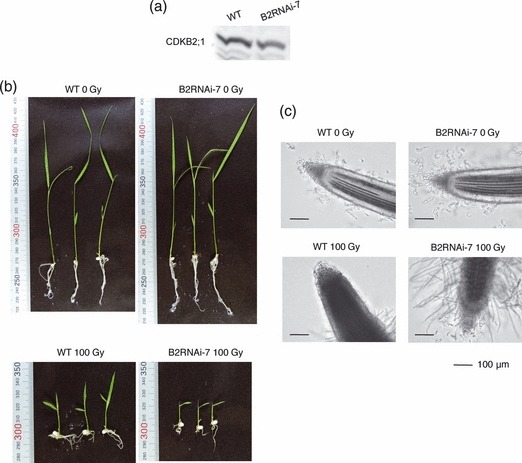
X-ray sensitivity of *Orysa;CDKB2;1* knockdown plants. (a) Western blot analysis of CDKB2;1. Crude protein was prepared from roots of non-irradiated wild-type (WT) and B2RNAi-7 plants. (b) WT and *CDKB2;1* knockdown plants (B2RNAi-7-1 and B2RNAi-16-1) grown on MS culture medium were irradiated with 100 Gy X-rays. Non-irradiated and irradiated plants were grown on MS culture medium for 5 days. (c) Morphology of primary root tip response with or without 100 Gy X-ray irradiation.

**Figure 7:**
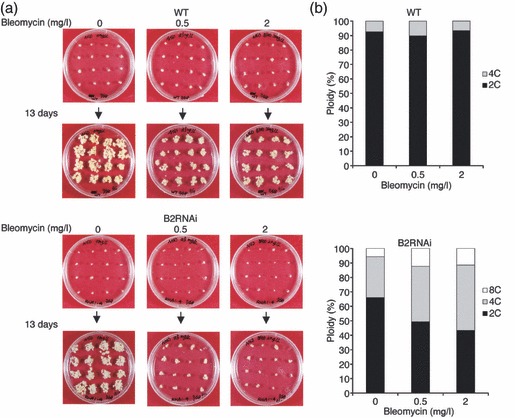
Effect of constitutive DNA damage by bleomycin on growth and ploidy of the calli. (a) Proliferation of calli on medium supplemented with 0, 0.5 or 2mg L^−1^ bleomycin. (b) Ploidy of rice calli grown on medium containing 0, 0.5 or 2 mg L^−1^ bleomycin for 10 days.

To investigate the effect of *CDKB2;1* knockdown on the DNA damage response, we analyzed the X-ray sensitivity of B2RNAi plants. Although the decrease in CDKB2;1 protein levels was not great (Figure 6a), and no growth inhibition was detected under normal conditions (Figure 6b, upper panel), B2RNAi-7 plants stopped growing almost immediately after irradiation (Figure 6b, lower panel). Furthermore, cells in the root tips of the X-ray-irradiated B2RNAi-7 mutant were swollen and disorganized. The profuse production of root hairs detected in X-ray-irradiated B2RNAi-7 plants is a typical morphological phenotypic response to DNA damage in roots (Hefner *et al.*, 2006). DNA damage hypersensitivity due to *CDKB2;1* knockdown was more marked when we used calli to analyze bleomycin sensitivity. In wild-type calli, bleomycin treatment (0.5 and 2 mg L^−1^) disturbed cell proliferation (Figure 7a), but these treatments did not induce polyploidy or accumulation of 4C nuclei (Figure 7b). These results indicate that G_2_ arrest or inhibition of mitosis is not the major DNA damage response in rice. However, in *CDKB2;1* knockdown calli, proliferation was severely inhibited (Figure 7a) and polyploidy was enhanced (Figure 7b) by bleomycin treatment. Endomitosis is due to inhibition of mitosis; *CDKB2;1* knockdown calli may be arrested at M phase due to accumulated DNA damage at M phase, and/or CDKB2 may be involved directly in the progression of M phase in rice. Application of bleomycin may further induce DNA damage at M phase in *CDKB2;1* knockdown callus, which may result in enhanced endomitosis.

In mammals and yeast, there are several reports on the function of cell-cycle regulators in DNA repair. Cyclin A1 expression is reported to be induced by γ-irradiation, and cyclins A1 and A2 enhance DNA DSB repair by homologous recombination in mice (Müller-Tidow *et al.*, 2004). Esashi *et al.* (2005) described CDK-dependent phosphorylation of BRCA2, which interacts directly with the essential recombination protein Rad51 in cultured human cells. Furthermore, involvement of CDK in recruitment of Rad51 to the site of DNA DSBs has been demonstrated in *Saccharomyces cerevisiae* (Aylon *et al.*, 2004; Ira *et al.*, 2004). In contrast, the function of cell cycle-related factors in the DNA damage response in plants is not clear. CDKB2;1, which is a plant-specific CDK expressed only in G_2_/M phase, may have a function in enhancing DNA repair at this stage in rice. Alternatively, impaired cell-cycle progression may increase cell sensitivity to DNA damage.

### Concluding remarks

In contrast to Arabidopsis, which shows increased DNA ploidy in response to DNA DSBs or *CDKB2* knockdown, no induction of endoreduplication or *CDKB2;1* down-regulation was detected following DNA damage in rice. We speculate that these differences are related to different strategies for morphogenesis and cell survival in response to DNA damage. In Arabidopsis, DNA replication and cell division occur not only in meristematic tissues but also in other tissues (Donnelly *et al.*, 1999). Furthermore, pavement cells of Arabidopsis leaves have a variety of sizes and shapes and form a mosaic-like structure. These morphological flexibilities may allow Arabidopsis to utilize endoreduplication in order to avoid cell death by DNA damage. In contrast to Arabidopsis, growth of rice leaves results from cell proliferation at the base of the leaf and cell elongation that takes place in the elongation zone directly above the meristem. This spatial gradient, synchronized cell division and cell expansion results in leaf growth taking place predominantly along a one-dimensional axis. Disorganized cell elongation accompanied by endoreduplication in rice would disturb normal morphogenesis and development. Thus, rice cells may need to suppress entry to the endocycle except in the endosperm.

Previously published evidence has shown that meristem cells respond differently to DNA damage compared with differentiated cells. In Arabidopsis, DNA DSBs cause cell-cycle arrest at G_1_/S in meristem cells but not in endoreduplicating differentiating cells (Hefner *et al.*, 2006). Root and shoot stem cells and their early descendants are killed selectively by treatment with radiomimetic drugs, X-rays or mutations that disrupt DNA repair by non-homologous end-joining (Fulcher and Sablowski, 2009). In addition, within both the root and shoot meristem, ataxia telangiectasia mutated (ATM) and ATM/RAD3-related (ATR) protein kinase-dependent, non-apoptotic programmed cell death is used to eliminate damaged cells specifically from the population of stem cells and their early descendants (Fulcher and Sablowski, 2009). These results indicate that the response to DNA damage is cell type-dependent, and that endoreduplication is repressed in stem cells of Arabidopsis. By contrast, endoreduplication in rice plants may be actively repressed not only in stem cells but also in meristematic cells. Thus rice may not deploy mechanisms to induce endoreduplication upon DNA damage, but instead may induce early onset of cell differentiation as well as extensive repair of damaged DNA during cell-cycle arrest.

In this study, we did not detect *Orysa;CDKB2;1* down-regulation in response to DNA damage. DNA damage responses are mediated by the highly conserved ATM and ATR protein kinases (Shiloh, 2006; Su, 2006; Cimprich and Cortez, 2008). Furthermore, a plant-specific transcription factor, SOG1 (suppressor of gamma response 1) has been shown to process signals associated with multiple responses to DNA damage, and to function downstream of ATM and ATR (Yoshiyama *et al.*, 2009). Recently, Adachi *et al.* (2011) demonstrated that expression of *Arath;CDKB2;1* was suppressed in response to DSBs at both the transcriptional and protein level, but this down-regulation was not necessarily required for endocycle induction because treatment with the DNA-damaging agent zeocin did not decrease the protein level of CDKB2;1 in root tips of an Arabidopsis *atr* mutant although endoreduplication occurred normally. These results indicate that reduction of the *CDKB2* level is one of the factors inhibiting entry into M phase, but that other factors also contribute to down-regulating mitotic CDK activities, leading to endoreduplication in Arabidopsis. In summary, induction of DSBs up-regulates CDK inhibitors and down-regulates cyclin A/B and CDKB2 via the ATM/ATR–SOG1 signal transduction pathway, resulting in increased endoreduplication. Putative ATM and ATR homologs (Os01g0106700 and Os06g0724700, respectively) have been found in the rice genome database. However, the function of these protein kinases has not yet been analyzed. The non-degradable nature of *Orysa*;CDKB2;1 after DNA damage could be explained by differences in the ATM- and ATR-mediated DNA damage signal transduction pathway between rice and Arabidopsis. Alternatively, *Orysa*;CDKB2;1 itself may be resistant to protein degradation, in contrast to *Arath*;CDKB2;1.

Additionally, unlike *Arath*;CDKB2;1, we detected an enhanced level of *Orysa*;CDKB2;1 protein after DNA damage. Furthermore, *Orysa*;*CDKB2;1* knockdown plants showed increased sensitivity to DNA damage. These data suggest the important role of *Orysa*;CDKB2;1 in DNA repair. In mammals, there is increasing evidence that the catalytic activities of CDKs play a critical role in the DNA damage response (for review, see Yata and Esashi, 2009). It has been observed experimentally that chemical inhibitors of CDKs sensitize cells to reagents, such as ionizing radiation and cisplatin, that create DSBs (Ongkeko *et al.*, 1995; Maggiorella *et al.*, 2003; Deans *et al.*, 2006). Furthermore, analysis in yeasts and mammalian cells has revealed that CDK activity is essential for DNA resection and progression of homologous recombination repair during S and G_2_ phase (Caspari *et al.*, 2002; Ferreira and Cooper, 2004; Ira *et al.*, 2004; Deans *et al.*, 2006; Sonoda *et al.*, 2006). Remarkably, in most Arabidopsis mutants suffering from endogenous DNA stress, the *CYCB1:1* gene encoding a G_2_/M phase-specific B-type cyclin is strongly induced; this gene is also induced by DSB-causing treatments (Chen *et al.*, 2003; Culligan *et al.*, 2006; Ricaud *et al.*, 2007). This transcriptional induction depends on ATM. Interestingly, in ATR knockout plants, ATM-dependent *CYCB1;1* induction is apparent, but the protein shows decreased stability, suggesting that ATR controls its abundance post-transcriptionally (Culligan *et al.*, 2006). The role of CycB1;1 in the DNA stress response is unknown, but the unique behavior of CYCB1;1 hints at a specific function for this particular cyclin. Cools and De Veylder (2009) suggest that CYCB1;1 may block all cells from entering the endocycle, preventing loss of all division-competent cells, and allowing meristem reactivation after repair of DNA damage. We found that endomitosis occurs in *Orysa*;*CDKB2;1* knockdown cells. Interestingly, the presence of non-degradable CYCB1;1 in tobacco leads to a doubled DNA content as a result of endomitosis (Weingartner *et al.*, 2004). These results indicate that timely expression and degradation of mitotic cyclin and CDK in plants is required for reorganization of mitotic microtubules to the phragmoplast and for proper cytokinesis. In rice, CYCB1;1 has not been reported as the partner of CDKB2;1, but both CDKB2;1 and CYCB1;1 are expressed specifically during S/G_2_ phase. Further analyses are required to determine whether these factors work cooperatively in mitotic cell-cycle regulation, protein degradation after mitosis and the response to DNA damage.

## Experimental procedures

### X-ray irradiation

Rice calli on solid N6D medium were irradiated using an OM-100RS soft X-ray system (Omic) at a dose of 133 Gy/h. Exposure times of 2.5, 11 and 33 min are equivalent to irradiation of 5, 25 and 100 Gy, respectively.

### Comet assay

X-ray-irradiated rice calli grown on solid N6D medium were processed as described previously (N/N protocol; Menke *et al.*, 2001). A CCD camera was used to capture images of comets stained using SYBR Green (Lonza, http://www.lonza.com/group/en.html). Signals were quantified using Comet analyzer software (YOUWORKS, http://youworks.jp/) under conditions that excluded severely damaged nuclei. The intensity of DNA is shown in graded colors. DNA DSBs are represented as the ‘tail moment’ (Olive *et al.*, 1990), which is defined as the tail distance multiplied by the sum of tail intensity/sum of cell intensity. Individual parameters have been defined previously (Endo *et al.*, 2006). We analyzed 30–50 nuclei at each time point, and this experiment was repeated at least three times.

### Real-time PCR

Total RNA was prepared using an RNeasy plant mini kit (Qiagen, http://www.qiagen.com/) according to the manufacturer's instructions. The RNA preparation was then treated with RNase-free DNase I (Qiagen). First-strand cDNA synthesis was performed using oligo(dT) primer and ReverTra Ace (TOYOBO, http://www.toyobo.co.jp/). Detailed conditions for real-time PCR and the primer sequences used in this experiment are given in Appendix S1.

### Flow cytometric analysis

Nuclei extraction and DNA staining were performed using CvStain UV precise P (Partec, http://www.partec.com/). The filtered nuclei were subjected to flow analysis (Partec) with laser excitation at 357 nm.

### Western blot analysis

Proteins extracted from calli or liquid suspension-cultured cells were used for Western blot analysis. Proteins (20 μg) were fractionated by SDS–PAGE on a 5–20% Tris/glycine SDS gradient pre-cast polyacrylamide gel (Bio-Rad, http://www.bio-rad.com/), and subjected to immunoblotting using SuperSignal West Dura maximum sensitivity substrate (Thermo Scientific, http://www.piercenet.com/). Polyclonal antibody was raised in rabbits against the C-terminal peptide PYFNDVNKELY of *Os*CDKB2 (Umeda *et al.*, 1999b).

### *OsCDBK2* RNAi plasmid construction

The *OsCDBK2* RNAi plasmid was constructed as described in Appendix S1.

### Rice transformation

*Agrobacterium*-mediated transformation of rice (*Oryza sativa* L. cv. Nipponbare) was performed as described previously (Toki, 1997; Toki *et al.*, 2006). After co-cultivation of *Agrobacterium* carrying the binary vector with rice scutellum-derived calli (pre-cultured for 3 weeks) for 3 days, infected calli were transferred to fresh callus induction medium (Toki, 1997) containing 50 mg L^−1^ hygromycin B (Wako Pure Chemicals, http://www.wako-chem.co.jp/english/) and 35 mg L^−1^ carbenicillin (Wako) to remove *Agrobacterium*. Hygromycin-tolerant cells were selected over a period of 25 days.

### Scanning electron microscopy

Scanning electron micrographs of leaf epidermal cells (Figure 5c) were obtained using a VE-8800 scanning electron microscope (Keyence, http://www.keyence.com/) at 0.8 kV with a secondary electron detector.

### Northern blot analysis

Northern blot analysis was performed as described in Appendix S1.

### Chromosome observation

Chromosome observation was performed as described in Appendix S1.

## SUPPORTING INFORMATION

**Figure S1.** Effect of high dosage X-ray irradiation on the ploidy and expression of *CDKB2;1* in wild-type rice calli.

**Figure S2.** Alignment of rice *CDK* mRNAs.

**Figure S3.** Transcription of *CDK* genes in *Orysa;CDKB2;1* knockdown rice calli.

**Figure S4.** Increased chromosome number in B2RNAi-10.

**Figure S5.** *Orysa;CDKB2;1* knockdown lines with increased ploidy levels cease growth after regeneration.

**Figure S6.** *Orysa;CDKB2;1* inducible knockdown can induce G_2_ arrest.

**Appendix S1.** Methods for real-time PCR, plasmid construction, Northern blot analysis and chromosome observation.

Please note: As a service to our authors and readers, this journal provides supporting information supplied by the authors. Such materials are peer-reviewed and may be re-organized for online delivery, but are not copy-edited or typeset. Technical support issues arising from supporting information (other than missing files) should be addressed to the authors.
