# Supplementary figures and images for: CDKB2 is involved in mitosis and DNA damage response in rice
Source: Plant J. 2011 Dec 15;69(6):967–77. doi: 10.1111/j.1365-313X.2011.04847.x (PMC3440594; doi:10.1111/j.1365-313X.2011.04847.x)

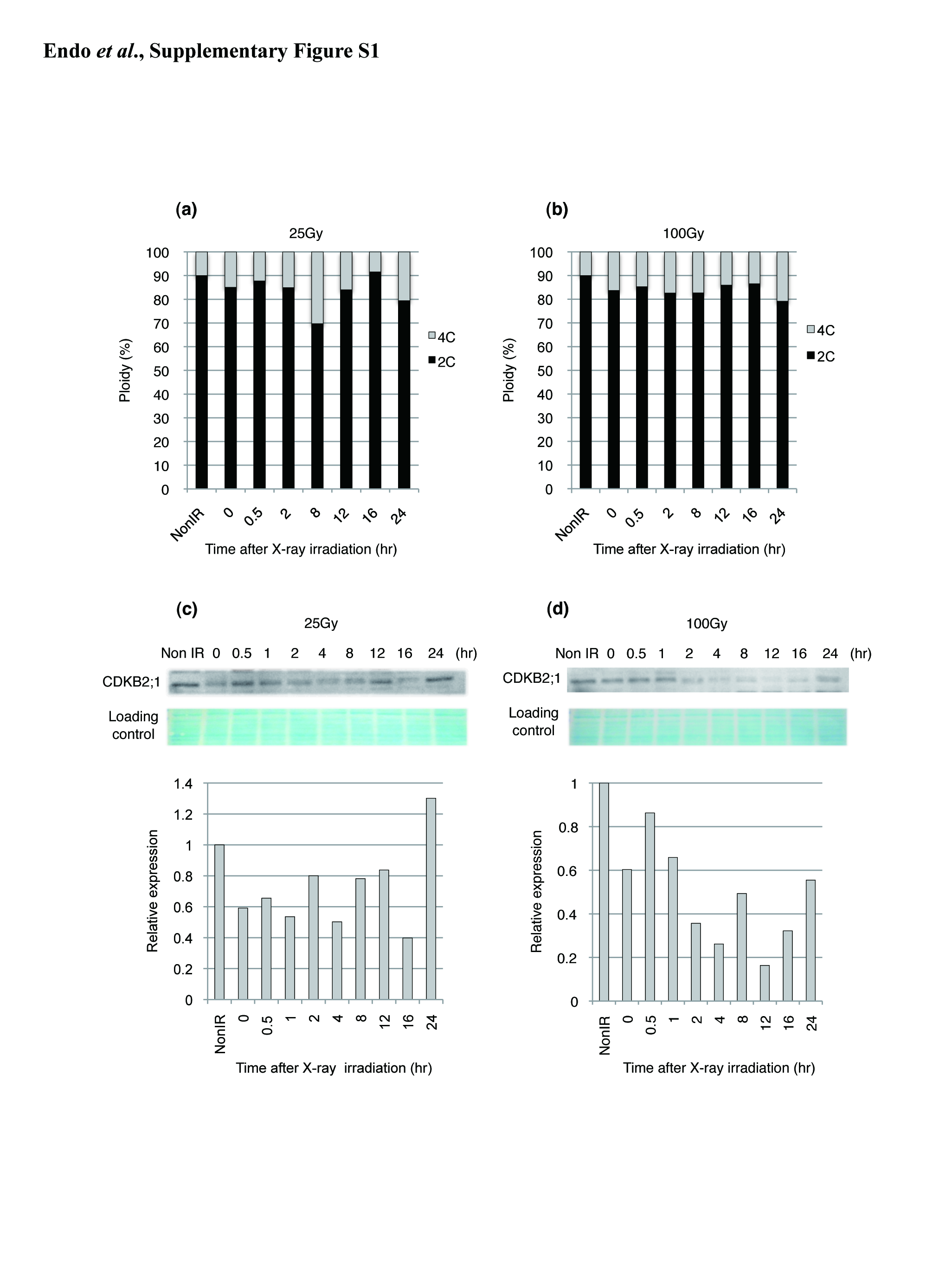

Supplement: Supplementary file 1 [file tpj0069-0967-SD1.tif]

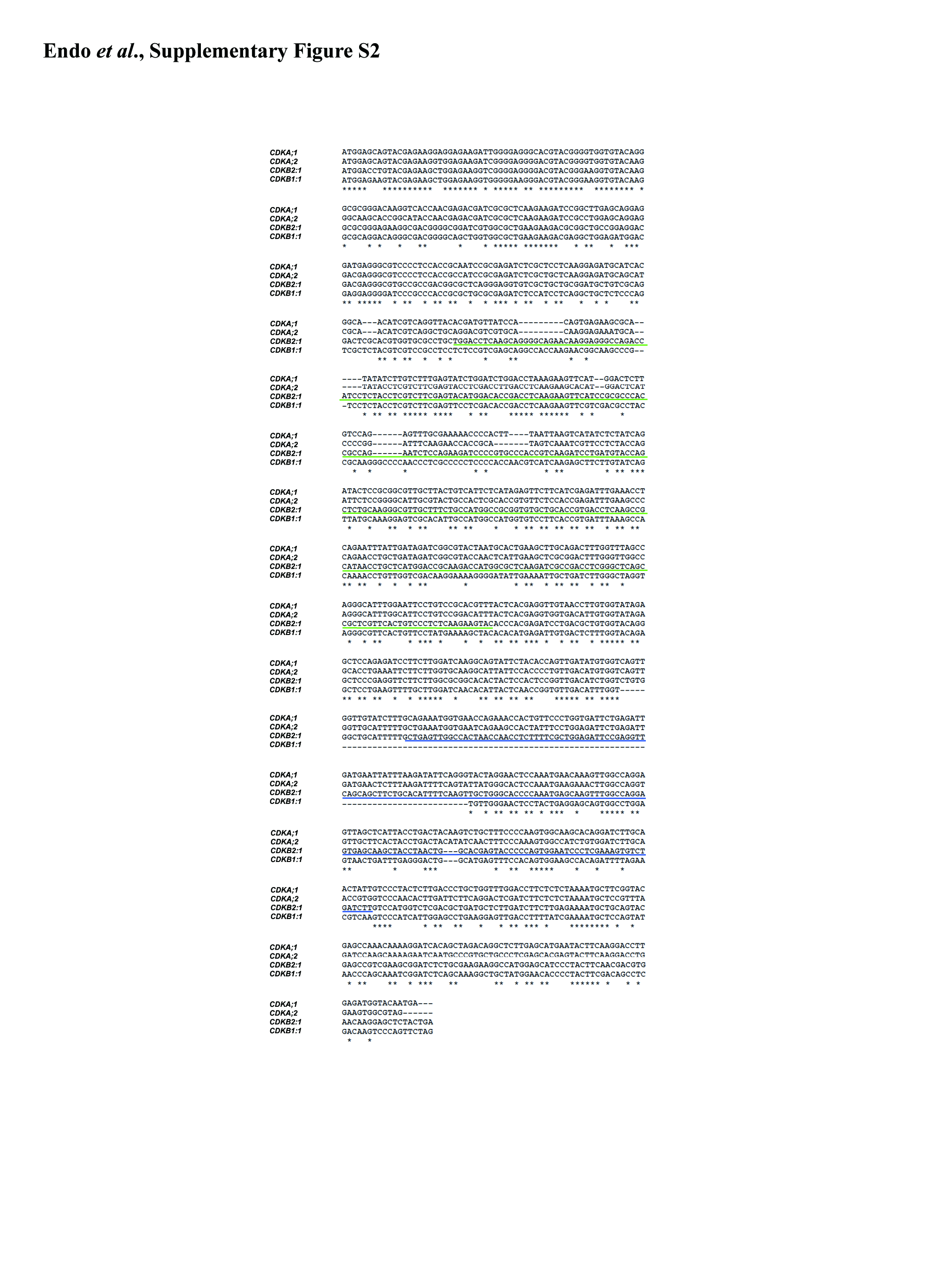

Supplement: Supplementary file 2 [file tpj0069-0967-SD2.tif]

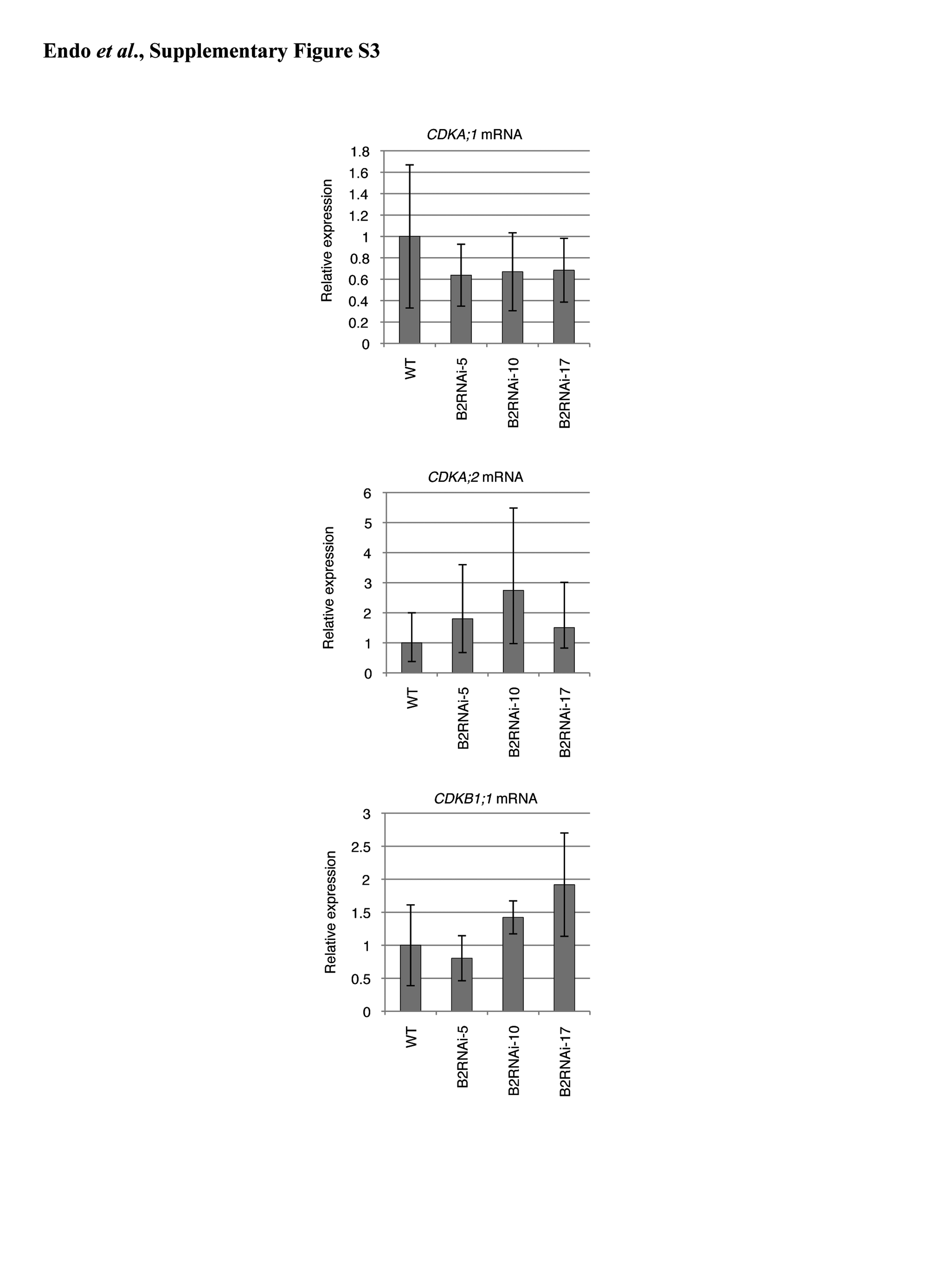

Supplement: Supplementary file 3 [file tpj0069-0967-SD3.tif]

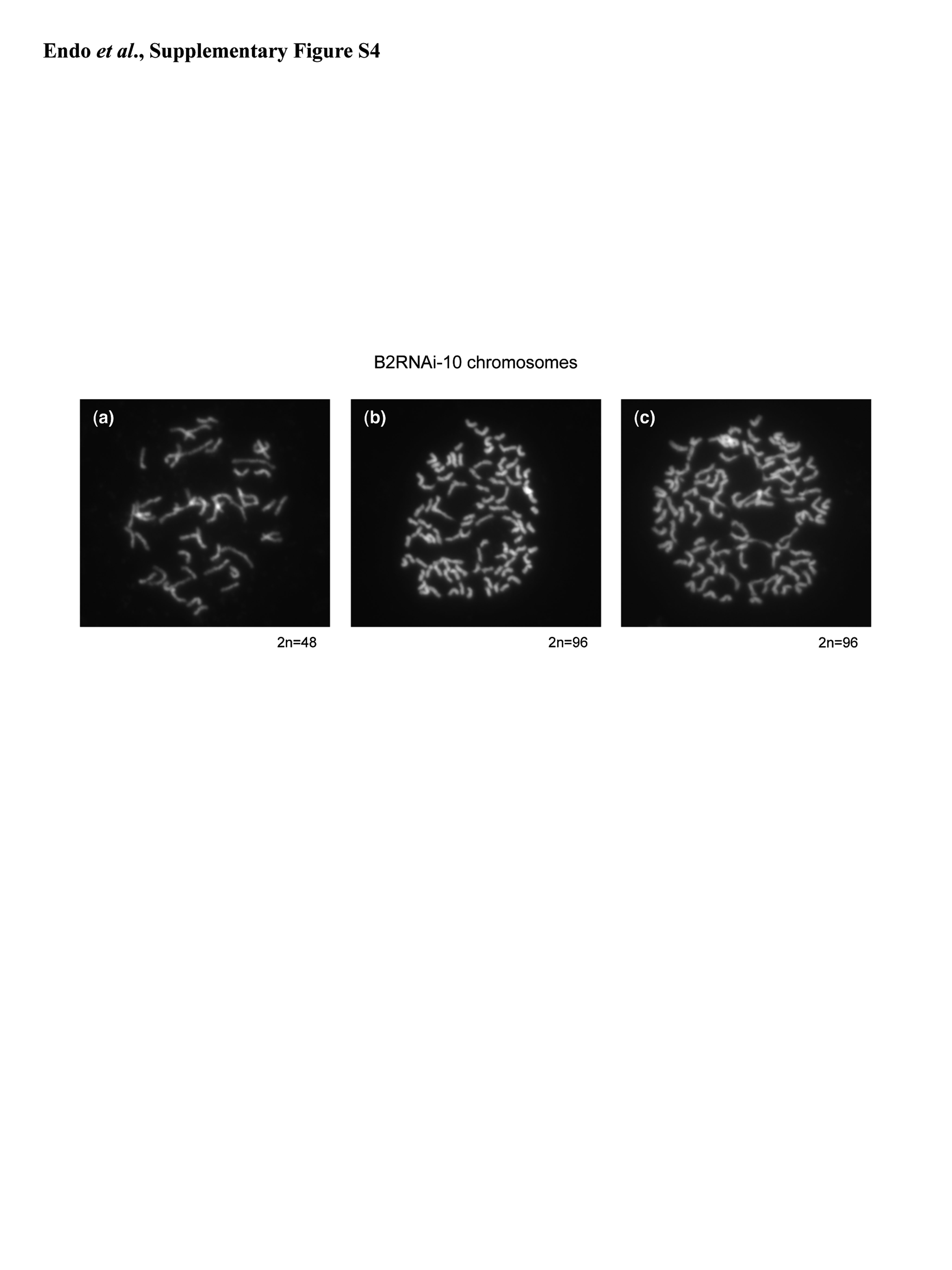

Supplement: Supplementary file 4 [file tpj0069-0967-SD4.tif]

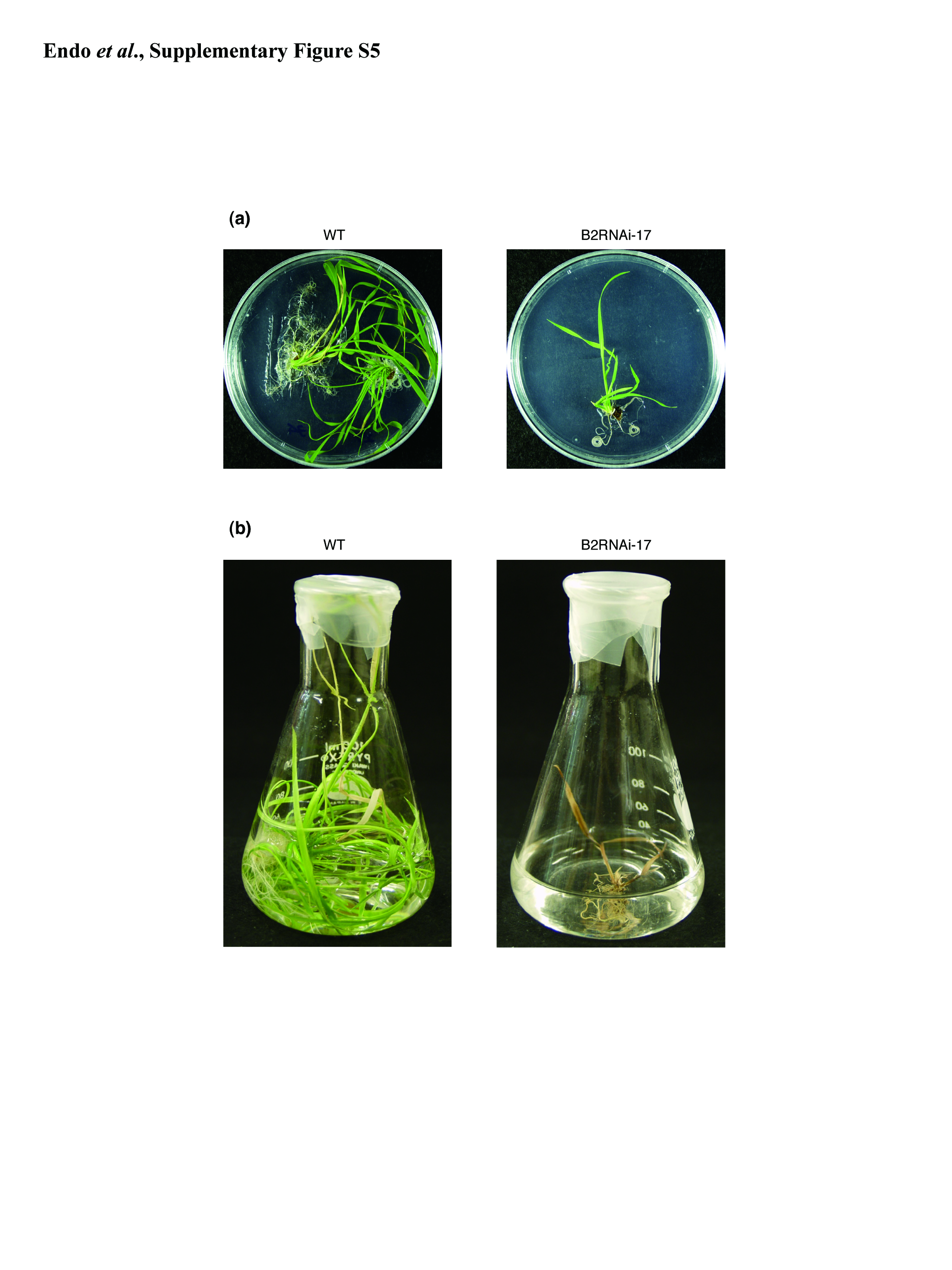

Supplement: Supplementary file 5 [file tpj0069-0967-SD5.tif]

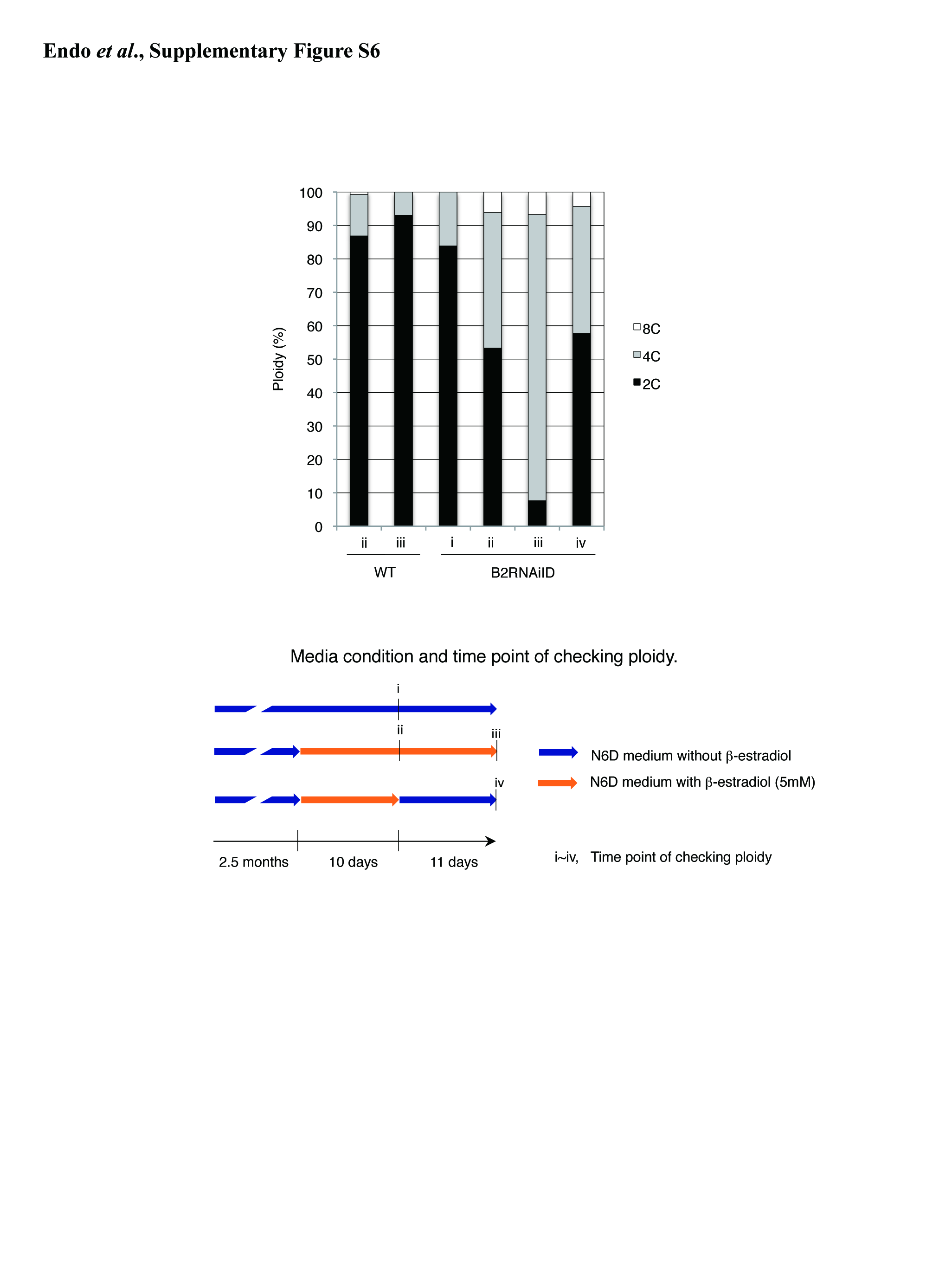

Supplement: Supplementary file 6 [file tpj0069-0967-SD6.tif]
