# Supplementary material for: CDKB2 is involved in mitosis and DNA damage response in rice
Source: Plant J. 2011 Dec 15;69(6):967–77. doi: 10.1111/j.1365-313X.2011.04847.x (PMC3440594; doi:10.1111/j.1365-313X.2011.04847.x)
Supplement: Supplementary file 7 [file tpj0069-0967-SD8.doc]

**Figure legends to Supplementary Figures**

**Figure S1** Effect of high dosage X-ray irradiation on the ploidy and expression of CDKB2;1 in wild-type rice calli.

Ploidy of calli after 25 Gy (a) and 100 Gy (b) X-ray irradiation. CDKB2;1 protein levels in 25 Gy (c) and 100 Gy (d) irradiated calli .

**Figure S2** Alignment of rice *CDK mRNAs*.

The green underlined sequence shows the RNAi region of *Orysa;CDKB2;1*. The blue underlined sequence shows the probe region used for Northern blot analysis. This RNAi region shares 55.8%, 59.7%, 67.8% homology with *Orysa;CDKA;1, Orysa;CDKA;2* and *Orysa;CDKB1;1* respectively.

**Figure S**3 Transcription of *CDKs* in *Orysa;CDKB2;1* knockdown rice calli.

Transcription level of *Orysa;CDKA;1*, *Orysa;CDKA;2*, *Orysa;CDKB1;1* as determined by real-time quantitative PCR. Detailed conditions used for real-time quantitative PCR are given in Supplementary methods.

**Figure S4** Increased chromosome number observed in B2RNAi-10.

We detected nuclei with 48 (a) or 96 (b, c) chromosomes, but none with 24, in B2RNAi-10 calli, which contains 4C, 8C and 16C, but no 2C in ploidy analysis (Figure 3d).

**Figure S5** *Orysa;CDKB2;1* knockdown lines with increased ploidy level cease growth after regeneration.

Calli that grew vigorously on selection medium were transferred to regeneration medium. Regenerated plants were further grown on hormone-free medium to facilitate root growth. (a) Regenerated plants of B2RNAi-17, which showed increased ploidy level in calli, ceased growth on hormone-free medium. (b) The B2RNAi-17 regenerated plant died, even following removal of the physical impedance to root growth by using liquid hormone-free medium. Photos were taken 12 days after transfer to liquid hormone-free medium.

**Figure S6** *Orysa;CDKB2;1* inducible knockdown can regulate G2 arrest.

Calli of wild-type (WT) and *CDKB2;1* inducible knockdown lines (B2RNAiID) were transferred to fresh N6D medium with (+) or without (-) 5mM β-estradiol. Ten days after transfer, we analyzed ploidy (i), (ii). An increased 4C population and the emergence of an 8C population in β-estradiol-treated B2RNAiID calli (ii) indicates that inducible knockdown of *CDKB2;1* leads to G2 arrest and/or endomitosis. When β-estradiol treatment was extended to 21 days (iii), the increase in the 4C population was enhanced. When B2RNAiID calli on N6D medium with β-estradiol were re-transferred to N6D medium without β-estradiol (iv), enhancement of polyploidy ceased.
