# Supplementary material for: CDKB2 is involved in mitosis and DNA damage response in rice
Source: Plant J. 2011 Dec 15;69(6):967–77. doi: 10.1111/j.1365-313X.2011.04847.x (PMC3440594; doi:10.1111/j.1365-313X.2011.04847.x)
Supplement: Supplementary file 8 [file tpj0069-0967-SD7.doc]

**Supplementary Methods**

***Real-time PCR***

Real-time PCR was performed in a total volume of 10 µl using 1 µl of cDNA as a template, 200nM (*OsRAD51A2, Orysa;CDKA;1, Orysa;CDKA;2*) or 100nM (*Orysa;CDKB2;1, Orysa;CDKB1;1*) primers and 5 µl of 2x Power SYBR Green PCR Master Mix (Applied Biosystems, Foster City, CA, USA). The following primers were used for real-time PCR analysis:

*Orysa;CDKA;1* forward 5’-GGACAAGGTCACCAACGAGACG-3’,

*Orysa;CDKA;1* reverse 5’-TAACCTGACGATGTTGCCGTGATG-3’;

*Orysa;CDKA;2* forward 5’-CAAGCACCGGCATACCAACG-3’,

*Orysa;CDKA;2* reverse 5’-CAGCCTGACGATGTTGCGATGCTG-3’;

*Orysa;CDKB1;1* forward 5’-GTAACTGATTTGAGGGACTGGCATGAG-3’,

*Orysa;CDKB1;1* reverse 5’- AGCCTTTGCTGAGATCCGATTTGCT-3’;

*Orysa;CDKB2;1* forward *5’-*GCAGCTTCTGCACATTTTCAAG-3’,

*Orysa;CDKB2;1* reverse 5’- TGCTCACTCCTGGCCAAACT-3’;

*OsRAD51A2* forward 5’-TGGTGGACGCTTGGATTGAT-3’,

*OsRAD51A2* reverse 5’-CAGGATTCCAGGGCGCTAT-3’;

*OsACT1* forward 5’-AGGCCAATCGTGAGAAGATGACCCA-3’,

*OsACT1* reverse 5’-GTGTGGCTGACACCATCACCAGAG-3’

An ABI PRISM 7000 (Applied Biosystems) was used to perform PCR cycles, and fluorescence was quantified against standards. cDNAs were amplified under the following conditions; i) 95°C for 10 sec for one cycle; ii) 95°C for 5 sec, iii) 60°C for 1 min for 40 cycles. Melting temperatures were estimated for every gene product. *OsACT1* was used as an internal control. The average of 3 reactions was used to calculate the fold induction.

***Plasmid construction of Orysa;CDBK2;1 constitutive RNAi (B2RNAi)***

The RNAi region was amplified by PCR using plasmid DNA carrying the *Orysa;CDKB2;1* ORF as a template and the following primer pairs:

cdc2OS3-RNAi-for+attB1 5’-AAAAAAGCAGGCTTGGACCTCAAGCAGGGGC-3’, cdc2OS3-RNAi-rev+attB2 5’-AAGAAAGCTGGGTGTACTTCTTGAGAGGGACAG-3’.

These PCR products were used as templates for a second PCR with the following primer pairs:

attB1-adapter 5’-GGGGACAAGTTTGTACAAAAAAGCAGGCT-3’

attB2-adapter 5’-GGGGACCACTTTGTACAAGAAAGCTGGGT-3’

Second PCR products were subcloned into pDONR221 to yield an entry vector by a BP clonase-catalyzed reaction (Invitrogen, Carlsbad, California). The final RNA silencing binary vectors were produced in an LR clonase-catalyzed reaction (Invitrogen) between pDONR221 and the pANDA vector (Miki and Shimamoto, 2004) for transformation of rice calli. After introduction of the *Orysa;CDKB2;1* RNAi construct into wild-type rice calli (cv. Nipponbare), we obtained several independent T_0_ transgenic calli (B2RNAi).

***Plasmid construction of Orysa;CDKB2;1 inducible RNAi (B2RNAiID)***

After digestion of the *Orysa;CDKB2;1* constitutive knockdown construct with *Apa*I and *Spe*I, the RNAi silencing cassette carrying inverted repeats of *Orysa;CDKB2;1* fragment with GUS linker was subcloned into the *Apa*I/*Spe*I sites of the estrogen-inducible vector pER8 (Zuo *et al.*, 2000).

***Northern blot analysis***

Total RNA was prepared using an RNeasy Plant Mini Kit (Qiagen). Total RNA (10 µg) was loaded and separated on a 1% agarose gel and transferred onto a positively charged nylon membrane (Roche, Basel, Switzerland). The probe was prepared using a PCR DIG probe synthesis kit (Roche) with PCR primers *Orysa;CDKB2;1*-F3 5’-TGCTGAGTTGGCCACTAACCAACCTCT-3’, and *Orysa;CDKB2;1*-R3 5’-AAGATCAGACACTTTCGAGGGA-3’.

Hybridization was performed according to the DIG Application Manual (Roche). Hybridization was at 48°C and washing was performed under high-stringency conditions at 50°C.

***Chromosome observation***

Procedures for chromosome observation were modified from methods previously described for *Arabidopsis* (Abe *et al.,* 2005). Calli with an average size of 5 mm in diameter were fixed in freshly prepared fixative (3:1 methanol-acetic acid mixture) at 4°C for several days without any pretreatment. Next, these calli were washed in de-ionized water for 30 min twice in a Petri dish, and the calli were macerated with two-fold diluted enzyme mixture at 37°C for 50–60 min. Macerated calli were then rinsed with de-ionized water for 10 min in the Petri dish. Each callus was laid onto a clean glass slide, and tapped with the tip of fine forceps into invisible particles in fresh fixative to spread the chromosomes. Staining and observation of chromosomal preparations were carried out as previously described (Abe *et al.,* 2005).

**References**

**Abe, K., Osakabe, K., Nakayama, S., Endo, M., Tagiri, A., Todoriki, S., Ichikawa, H. and Toki, S.** (2005) *Arabidopsis* RAD51C gene is important for homologous recombination in meiosis and mitosis. *Plant Physiol.* **139**, 896-908.

**Zuo, J.R., Niu, Q.W. and Chua, N.H.** (2000) An estrogen receptor-based transactivator XVE mediates highly inducible gene expression in transgenic plants. *Plant J.* **24,** 265-273.
